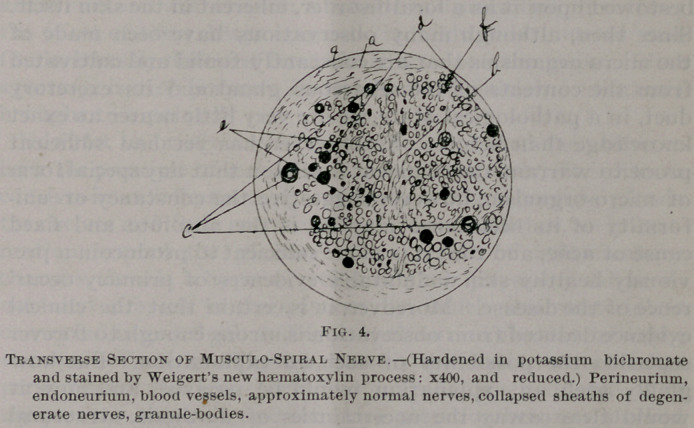# A Case of Multiple Neuritis

**Published:** 1895-08

**Authors:** H. F. Harris

**Affiliations:** Professor of Chemistry Southern Medical College; Pathologist to Grady Hospital, Atlanta, Ga.


MULTIPLE NEURITIS.

THE

### Southern Medical Record.

A MONTHLY JOURNAL OF MEDICINE AND SURGERY.

VOL. XXV. ATLANTA, GA., AUGUST, 1895. No. 8.

### Original Articles.

### A CASE OF MULTIPLE NEURITIS.

BY H. F. HARRIS, M. D.,

Professor of Chemistry Southern Medical College; Pathologist to Grady Hospital, Atlanta, Ga

As a result of the labors of Joffroy, Leyden, Stewart and Buzzard, the recognition of multiple neuritis is not now of such rare occurrence as formerly, but not so common, I hope, as to make without interest the record of a case presenting all the striking peculiarities of the disease.

The patient was first seen by me on Dec. 1st, 1892, at which time the following notes were taken:

Owing to the peculiar mental condition of the patient, which will be referred to later, neither his family nor personal history could be learned with any certainty previous to his coming to Georgia, about three years ago. His statements are, however, appended:

W. E. S., age 35, a male, and was born in Virginia. Family history good. When a child had mumps, measles, pleurisy and typhoid fever. Never had syphilis or rheumatism. He always smoked a great deal. Has used alcohol habitually since a child, and since manhood has been reached, has never taken less than two or three drinks daily,—often very much more. From outside sources it is learned that his habits have been very irregular for the last three years,—he working long hours, and his profession, being that of a pharmacist, afforded every facility for the taking of morphine and large quantities of whiskey, which he constantly did. At the time of the beginning of his trouble he was taking several grains of morphine and about a quart of whiskey daily. About two and one-half months before I first saw him, tingling in the extremities was noticed, followed by sharp, shooting pains which constantly grew worse; soon after the skin and muscles became exquisitely sensitive, eyesight failed and his memory rapidly became defective; locomotion became difficult, both on account of loss of power and extreme pain, and in a short time was lost altogether. Contemporaneously, marked mental derangement

FIG. 1.

LONGITUDINAL SECTION OF DEGENERATE MUSCLE FIBRES.—(Hardened in sublimate, and stained with hæmatoxylin and picric acid: x400, and reduced.) Showing loss of striation, increase of muclei and granular degeneration of muscle fibres.

manifested itself. No information could be obtained as to the severity of the inflammatory phenomena in the very beginning.

Paralysis first became marked in the flexors of the feet, followed by loss of power in the extensors, and later, paralysis of the extensors and flexors of the fingers, hands and forearms, in the order named. The muscles of the body were never involved to any appreciable extent.

During the following two months he remained perfectly helpless and suffered very greatly with pains all through the extremities; the skin and muscles continued extremely sensitive and he became very hysterical. The pulse was constantly rapid and weak, but the temperature rarely rose above the normal.

Present condition:—The heart and lungs are normal, as are also the spleen and liver so far as could be ascertained. The urine is normal. Tongue slightly coated. Temperature 99°F., pulse 95 and respiration 25. There is marked tenderness of hands, arms, feet and legs. Sensation to touch and pain delayed. The patient's peculiar mental condition rendered futile all attempts to gain accurate information respecting the distribution of the hyperæsthesia and even makes it impossible to distinguish with certainty between the phenomena of touch and pain.

The muscular weakness in the extremities is extreme,—often to the extent of complete paralysis—but varies with different muscles. There is total paralysis of the extensors of the left hand and fingers, and almost complete paralysis of those of the right. There is no grasping power in the left hand, but to a slight degree it is still retained in the right. Neither hand can be placed in the supine position. Flexion and extension of the forearms can still be accomplished with about equal power on both sides, but power is much diminished. The arms can be fully raised, but the power of carrying them to the sides seems normal.

Below the knees there is absolute paralysis. The flexors of the thigh are weak; the extensors scarcely retain any power.

The wrist drop which resulted from the paralysis of the extensors of the hand and the extension of the feet from palsy of their flexors, is well shown in the accompanying photograph.

All the deep reflexes are lost.

The muscles of the affected areas respond to very slight galvanic stimulation, but not at all to the strongest faradaic currents. The patient was so nervous, and the electrical applications caused so much pain, that no attempt was made to work out the qualitative changes; but there is no reason to doubt that the degenerative reaction was in all particulars complete.

The skin of the hands and feet is scaly and badly nourished. On the feet are many dark, congested areas, which closely resemble the discoloration that results from the application of silver nitrate to the denuded cuticle; he, however, denies having had, at any time, sores at these points. The nails are poorly nourished, and are very brittle.

The mental condition is quite characteristic. He moans and groans constantly, complains of great pain, and begs piteously any one who approaches, for morphine for its relief. A light touch on any part of his extremities will cause him to cry out; but if he is touched without his knowledge, often no attention is paid to it. And again, he will display quite as much emotion if he is deceived into the belief that he is being struck, as if it were really so. He scarcely remembers from one hour to another; often, one story is told in the morning, and something

FIG. 2.

LONGITUDINAL SECTION OF MUSCULO-SPIRAL NERVE.—(Hardened in potassium bichromate and stained by Weigert's new hæmatoxylin process: x400, and reduced.) All of the nerves are more or less degenerate, perineurium, granule-bodies, nerve-corpuscle.

entirely opposite in the afternoon. No anæmic, hysterical girl could be more nervous or excitable than he is.

Before I saw the patient, he was treated with strychnine and electricity, and, as might have been expected, without the slightest benefit. Despite the fact that these agents have been long considered by the best authorities valueless—nay, even hurtful—in recent lesions of the central nervous system, and in acute inflammations of nerves, their use, and faith in their efficacy is still persisted in by a large body of our profession, in all stages of diseases of this character. On account of its general tonic effect, strychnine may be often given in these diseases with advantage after the acute symptoms have subsided, but after a somewhat extensive experience with electricity, candor forces me to say that I have lost all faith in its curative powers, and that no striking and unmistakable example of its beneficial effect has ever come under my observation. It is true that I have seen paralysis, both of cerebral origin, and as a result of nerve lesions, improve while under electrical treatment, but I have also seen the same thing, quite as decided and rapid, without any treatment. Electricity may keep up, to a certain extent, the tone of a muscle, and thus, in a measure, prevent atrophy, but it is certainly unreasonable to suppose that it could in any way affect a central lesion, upon which the whole trouble depends. It strikes me that the fact is too often lost sight of that electricity is nothing more than a form of force, nearly related to heat and light, and inferior to both, I think, as a therapeutic agent.

After coming under my observation, the patient was given a tonic, massage was prescribed, and he was placed on a light, nutritious diet. Morphine was given only when absolutely necessary. No whisky was allowed. Mercury in small doses, and potassium iodide were both tried, but as no especial result could be noticed, their use was abandoned.

The patient slowly improved in every way; the pains diminished, the hyperæsthesia grew less and some power returned to the paralyzed parts. He seemed in a fair way to recover, when he was suddenly attacked with dysentery on the 10th of June, 1893, and died as the result on the 22d of the same month. I was out of town at the time of his death and did not return until about forty hours after. Immediately on my return I took from the body pieces of the paralyzed muscles and sections of all of the large nerves of the extremities. The spinal cord was opened, but it was decided that it was in a state of beginning decomposition, and was therefore not preserved. For the same reason no inspection of the internal organs was made. It is known that changes in nerves, which somewhat resemble those that follow neuritis, begin shortly after death, and progressively increase until dissolution takes place. It is very unfortunate that the necropsy should have been so long delayed, but as the nerves seemed in good condition it was determined to examine them microscopically anyway. They were rather harder than normal. Specimens of the muscles and nerves were preserved in potassium bichromate solutions and in Haidenhain's mercury-bichloride-salt solution. From the former, specimens were stained by Weigert's new hæmatoxylin method, and from the latter by carmine, carmine and aniline blue and by Van Giesen's acid fuchsin, picric acid and hæmatoxylin process; from both, stains were also made with hæmatoxylin in combination with picric acid, eosin and benzo-purpurin. While it is impossible to say how much of the change occurred after death, microscopical examination showed in the most beautiful manner precisely those appear-

FIG. 3.

TRANSVERSE SECTION OF SCIATIC NERVE.—(Hardened in potassium bicromate and stained by Weigert's new hæmatoxylin process: x400, and reduced.) Perineurium, endoneurium, blood vessels, nearly normal nerves, remains of degenerate nerves, regeneration of nerves.

ances which are known to result from inflammation of nerve tissues. The changes in the muscles are also quite characteristic; the fibres are at points granular, have lost their striation and have a large increase in the number of their nuclei; while this increase of the muscle-nuclei seems universal it is most marked around the edges of the areas of granular change. The fibres are narrowed—often irreulgarly—and there seems a slight increase of the connective tissue between some of them. There are no collections of small round cells. No fat is found in the muscle fibres. The changes above referred to are shown in Fig. 1.

The nerve degeneration varies much in different nerves, and also in different fasciculi of the same nerve, but on the whole the musculo-spirals of both sides have suffered most, with the sciatic following next in order. In the musculo-spiral but few fibres remain normal; the nerve contents are broken up at variable intervals and are collected together in irregular masses which frequently assume a more or less rounded or oblong form. These rounded bodies are frequently connected with each other by the remains of the axis-cylinder, around which, covering it more or less completely, generally remains a small amount of myelin. When stained by Weigert's method the myelin of the nerves, when preserved in anything like its nor-

FIG. 4.

TRANSVERSE SECTION OF MUSCULO-SPIRAL NERVE.—(Hardened in potassium bichromate and stained by Weigert's new hæmatoxylin process: x400, and reduced.) Perineurium, endoneurium, blood vessels, approximately normal nerves, collapsed sheaths of degenerate nerves, granule-bodies.

mal integrity, stains very dark, becoming less so as the tissues become less normal until finally the very degenerate nerves are not stained at all. When the degeneration is extreme the primitive sheaths collapse. In the more healthy nerves some of the fibres seem in a process of regeneration. The nerve-corpuscles are swollen, granular and take the stains poorly. Between the fibres there are a great number of granule bodies, varying from 10–15 m. m. in diameter. The endoneurium is irregularly thickened. The blood vessels are normal. Nothing abnormal is anywhere found in either the perineurium or epineurium further than occasional small collections of small round cells around blood vessels. The changes are shown in Figs. 2–4 inclusive.

## Figures and Tables

**Figure f1:**
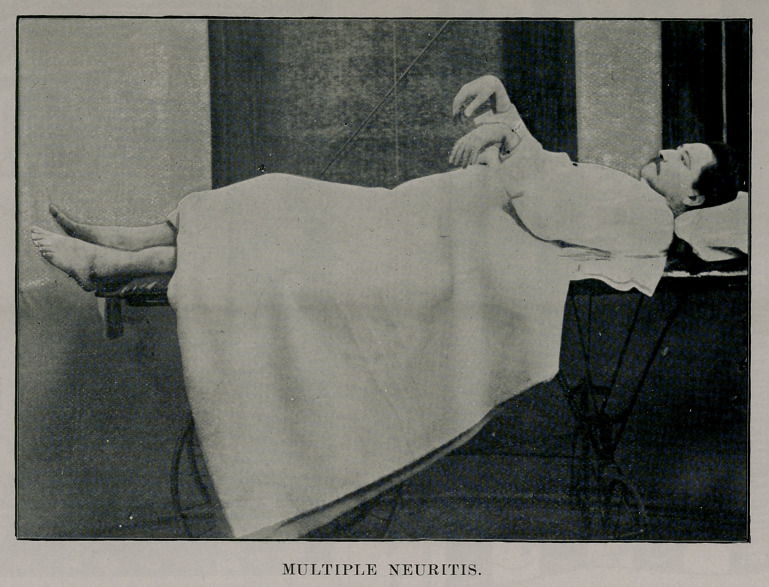


**Fig. 1. f2:**
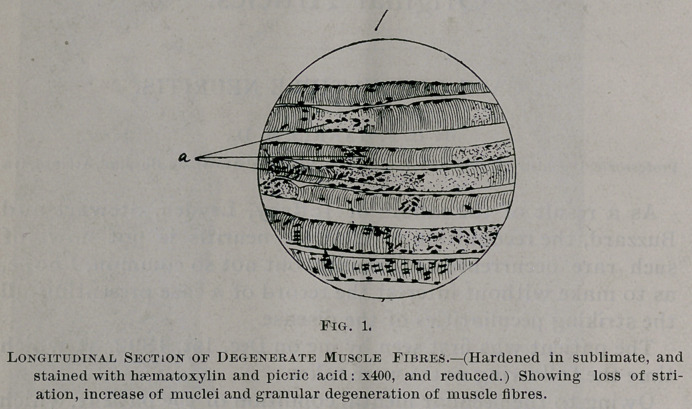


**Fig. 2. f3:**
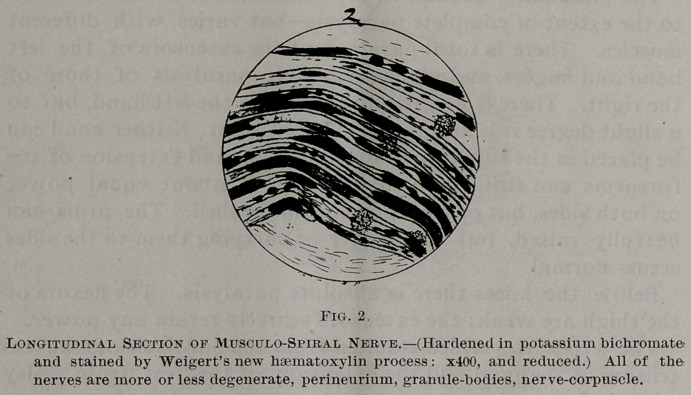


**Fig. 3. f4:**
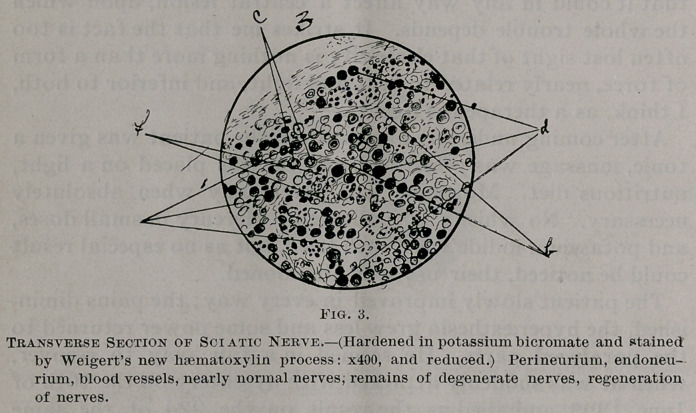


**Fig. 4. f5:**